# Blood biochemical profiles of Brahman crossbred cattle supplemented with different protein and energy sources

**DOI:** 10.14202/vetworld.2018.1021-1024

**Published:** 2018-07-31

**Authors:** Nguyen Hong Xuan, Huynh Tan Loc, Nguyen Trong Ngu

**Affiliations:** 1 Department of Food Technology, College of Food Technology and Biotechnology, Can Tho University of Technology, Can Tho, Vietnam; 2 Department of Veterinary Medicine, College of Agriculture and Applied Biology, Can Tho University, Can Tho, Vietnam

**Keywords:** cattle, concentrate, oil, soybean, supplementation

## Abstract

**Aim:**

The experiment was carried out to evaluate the effects of supplementing different levels of protein and energy sources on blood biochemical profiles of Brahman crossbred cattle.

**Materials and Methods:**

The study consisted of two experiments in Brahman crossbred cattle in An Giang Province. In trial 1, 28 cattle of 178±12.5 kg were arranged in a completely randomized block design. In the second trial, another 24 cattle of 182±14.3 kg were allocated in a 2 × 3 factorial design. The experiments lasted for 90 days. Blood samples were taken at the end of the experiments, and plasma concentrations of metabolites and enzymes were analyzed by an automated biochemical analyzer (Humalyzer 3000, USA).

**Results:**

The glucose concentration was highest at 1.83 mmol/L when supplemented with urea (60 g/head/d). Urea and creatinine content was not significantly different between treatments when cattle were supplemented with different protein and energy sources. In the treatment with 360 g/head/d soybean meal supplementation, cholesterol concentration was lowest (2.50 mmol/L), compared with the highest concentration (3.86 mmol/L) in the treatment with soybean meal at 720 g/head/day. The total protein concentration showed the highest values at 94.5 g/L and 96.3 g/L when supplemented with soybean meal (720 g/head/day) and fish oil, respectively.

**Conclusion:**

There were slightly altered blood biochemical profiles among cattle at different protein and energy source supplements.

## Introduction

Feeding and rearing systems play a pivotal role on the values of hematological and serum biochemical variables in raising animals [1-3]. Feed is an essential aspect of livestock production, and the importance of feed supplementation in animal production has substantially increased in recent years [4]. Nutritional status of an individual was dependent on dietary intake and effectiveness of metabolic processes, which can be determined using solely or a combination of chemical, anthropometric, biochemical, or dietary methods. In fact, a previous study has indicated that fish meal and soybean meal could be used as a source of protein in animal diets [5]. In addition, Hassan *et al*. [6] and Mamun *et al*. [3] stated that changes in biochemical and hematological constituents are major indicators of the physiological and pathological states of the animal.

Therefore, the biochemical determination of serum constituents and blood examination can provide valuable information relating to nutrition and other environmental factors that influence the performance and well-being of animals [7-9]. Serum concentrations of metabolites such as glucose, cholesterol, non-esterified fatty acids, blood urea nitrogen, creatinine, total proteins, albumin, globulin, and minerals are commonly used to assess the nutritional status of cattle.

The present study was conducted to explore the effects of different protein and energy sources on blood chemical profiles of Brahman crossbred cattle.

## Materials and Methods

### Ethical approval

This study was carried out after obtaining approval (No. 01/NCCB) from Can Tho University and An Giang Department of Veterinary Medicine.

### Animals and design

The study consisted of two experiments in Brahman crossbred cattle in An Giang Province, Vietnam (10°23’ N, 105°26’ E). In trial 1, 28 animals of 178±12.5 kg were arranged in a completely randomized block design. The basal diet consisted of *Hymenachne acutiglum* grass, rice straw, and rice bran (1.5 kg/head/d), and other treatments were with different protein sources and levels such as urea (U1, 60 g/head/d, and U2, 120 g/head/d), soybean meal (SM1, 360 g/head/d, and SM2, 720 g/head/d), and 50% blood meal and 50% feather meal mixture (BFM1, 360 g/head/d, and BFM2, 720 g/head/d). In the second trial, 24 cattle of 182±14.3 kg were allocated into a 2 × 3 factorial design with two levels of concentrate supplement (0.5% and 1.5% body weight) and three supplemental sources (no oil, 60 g soybean oil/kg dry matter [DM], and 60 g fish oil/kg DM). The resulting six treatments were 0.5% concentrate supplement without oil, 1.5% concentrate supplement without oil, 0.5% concentrate plus soybean oil supplement, 1.5% concentrate plus soybean oil supplement, 0.5% concentrate plus fish oil supplement, and 1.5% concentrate plus fish oil supplement. The experiments lasted for 90 days.

### Blood collection and analysis

At the end of the feeding trials, about 10 ml of blood samples were collected into tubes containing 10% sodium heparin. Samples were then centrifuged at 1000 rpm for 20 min at 4°C, and plasma was separated for laboratory analysis. The plasma concentrations of metabolites (glucose, urea, creatinine, uric acid, bilirubin, and cholesterol), protein (total protein, albumin, and globulin), and enzymes (aspartate aminotransferase and alanine aminotransferase [ALT]) were analyzed by an automatic biochemical analyzer (Humalyzer 3000, USA).

### Statistical analysis

Data were subjected to the analysis of variance in Minitab 16 Statistical Software using appropriate models as in the experimental design, and Tukey’s multiple test was used to determine the differences between means at the significant level of p<0.05 [10].

## Results

Table-1 shows that the differences between protein source supplementations also altered the cattle’s blood biochemical profiles. Glucose concentration was reduced in all treatments compared with basal treatment, except an increased glucose concentration in U1 treatment; glucose concentration in U1 treatment was higher than SB1, and the difference was statistically significant (p<0.05). Total protein concentration of all treatments was higher than that of the basal treatment. In SB2 treatment, the concentration was higher than that in basal treatment, and it was significantly different (p<0.05). There was a change in the composition of total bilirubin and direct bilirubin in U1 treatment at 29.67 μmol/L and 15.7 μmol/L and 5.23 μmol/L and 2.07 μmol/L higher than in SB1 treatment, respectively. This distinction was statistically significant (p<0.05). Cholesterol concentration also varied between treatments. In SB2 treatment, the cholesterol concentration was 3.86 mmol/L higher than that of the basal treatment with 3.43 mmol/L, and the difference was statistically significant (p<0.05). Urea concentration slightly increased in the treatments compared with the basal treatment, except a decrease in U1 treatment. However, the difference was not statistically significant.

**Table-1:** Enzyme activities and concentrations of proteins and metabolites in blood of cattle supplemented with different protein sources and levels (n=28)*.*

Parameters	n	Diets							SEM	p-value

		Basic	U1	U2	SB1	SB2	BFM1	BFM2		
Metabolites										
Glucose (mmol/L)	28	1.67^ab^	1.83^a^	1.73^ab^	0.57^b^	1.55^ab^	1.31^ab^	0.73^ab^	0.258	0.014
Urea (mmol/L)	28	4.47	4.33	4.47	5.83	5.35	7.00	6.40	0.621	0.061
Creatinine (µmol/L)	28	63.67	63.67	68.00	69.00	78.25	73.99	61.67	5.403	0.355
Uric acid (µmol/L)	28	61.67	156.33	49.67	55.67	64.75	171.33	73.00	34.012	0.128
Total bilirubin (µmol/L)	28	8.33^ab^	29.67^a^	11.3^ab^	5.23^b^	16.55^ab^	15.19^ab^	9.48^ab^	4.774	0.042
Direct bilirubin (µmol/L)	28	4.83^ab^	15.7^a^	7.50^ab^	2.07^b^	5.39^ab^	6.62^ab^	2.70^b^	2.664	0.039
Indirect bilirubin (µmol/L)	28	3.50	13.63	3.80	3.18	11.21	8.53	6.77	2.634	0.072
Cholesterol (mmol/L)	28	3.43^b^	3.73^ab^	3.83^ab^	2.50^b^	3.86^a^	3.46^ab^	4.97^ab^	0.308	0.002
Protein										
Total protein (g/L)	28	81.0^b^	90.0^ab^	90.0^ab^	89.3^ab^	94.5^a^	90.6^ab^	89.0^ab^	2.133	0.017
Albumin (g/L)	28	40.0	41.0	39.3	39.7	41.8	39.9	40.7	1.639	0.946
Globulin (g/L)	28	41.0^b^	49.0^ab^	50.7^ab^	53.0^a^	53.3^a^	50.5^ab^	48.3^ab^	2.452	0.042
Enzymes										
AST (U/L)	28	37.7	27.3	31.3	30.7	24.8	19.3	26.7	4.322	0.215
ALT (U/L)	28	18.0	14.7	20.7	20.7	26.5	13.8	17.3	2.798	0.091

Basic diet: *Hymenachne acutiglum* grass, rice straw, and rice bran (1.5 kg/head/d); U1, U2, SB1, SB2, BFM1, and BFM2: Supplements of urea (U1, 60 g/head/d, and U2, 120 g/head/d), soybean meal (SM1, 360 g/head/d, and SM2, 720 g/head/d), and 50% blood meal and 50% feather meal mixture (BFM1, 360 g/head/d, and BFM2, 720 g/head/d). SEM=Standard error of the means, AST=Aspartate aminotransferase, ALT=Alanine aminotransferase. ^a,b^Means bearing different superscripts within a row differ significantly (p<0.05)

Supplementing different concentrate levels and energy sources also changed the blood biochemical profiles of cattle (Table-2). In treatments without soybean oil and fish oil supplementation, the uric acid concentration was highest at 193.5 μmol/L and significantly different (p<0.05) compared with fish oil treatment, soybean oil treatment, and treatment at 0.5% concentrate supplement level. The concentration of total bilirubin and indirect bilirubin was also highest in treatments without soybean oil and fish oil, but there was no difference compared with the other treatments. Cholesterol concentration slightly increased from 3.3 to 3.7 mmol/L among treatments, and there was no statistically significant difference. The total protein concentration of fish oil treatment was 96.3 g/L, higher than that in treatment with soybean oil supplementation at 85.3 g/L, and the difference was statistically significant (p<0.05). However, the concentration of ALT in fish oil supplementation at 13.5 U/L was 19.3 U/L, less than that in soybean oil treatment (p<0.05).

**Table-2:** Enzyme activities and concentrations of proteins and metabolites in blood of cattle supplemented with different concentrate levels and energy sources (n=24).

Parameters	n	Concentrate (C)		SEM	Energy source (E)			SEM	p-value		
		
		0.5 (%)	1.5 (%)		-	SO	FO		C	E	CxE
Metabolites											
Glucose (mmol/L)	24	1.3	1.5	0.1	1.7	1.3	1.3	0.2	0.132	0.112	0.635
Urea (mmol/L)	24	5.8	5.7	0.2	5.3	5.9	6.1	0.3	0.789	0.120	0.179
Creatinine (µmol/L)	24	69.3	67.3	3.8	64.2	72.0	68.8	4.7	0.728	0.521	0.045
Uric acid (µmol/L)	24	97.7^b^	145.2^a^	11.3	193.5^A^	67.7^B^	103.0^B^	13.9	0.012	0.000	0.000
Total bilirubin (µmol/L)	24	10.3	16.7	3.9	23.5	7.8	9.3	4.7	0.257	0.069	0.031
Direct bilirubin (µmol/L)	24	9.5	9.2	1.5	13.0^A^	4.1^B^	4.9^B^	1.8	0.102	0.008	0.017
Indirect bilirubin (µmol/L)	24	4.8	7.6	2.5	10.5	3.7	4.4	3.0	0.440	0.257	0.053
Cholesterol (mmol/L)	24	3.7	3.3	0.1	3.5	3.6	3.3	0.2	0.070	0.484	0.784
Protein											
Total protein (g/L)	24	87.0	91.8	2.1	86.8^AB^	85.3^B^	96.3^A^	2.6	0.128	0.021	0.682
Albumin (g/L)	24	38.5^B^	41.3^A^	0.7	42.5^A^	39.0^B^	38.3^B^	0.8	0.013	0.009	0.278
Globulin (g/L)	24	48.5	50.5	2.1	44.3^B^	46.3^B^	58.0^A^	2.6	0.522	0.006	0.939
Enzymes											
AST (U/L)	24	30.0	28.7	2.1	30.5	25.8	31.8	2.6	0.667	0.271	0.016
ALT (U/L)	24	16.2	18.3	1.2	19.0^AB^	19.3^A^	13.5^B^	1.5	0.238	0.032	0.011

SO=Soybean oil, FO=Fish oil, C=Concentrate, E=Energy source, -=No supplementation of either soybean oil or fish oil, Means bearing different superscripts within a row differ significantly (p<0.05) (^a,b^Concentrate level, ^A,B^energy sources). SEM=Standard error of the means, AST=Aspartate aminotransferase, ALT=Alanine aminotransferase

## Discussion

The concentration of blood glucose content in the blood biochemical profiles was 0.57 nmol/L and 1.83 nmol/L for SB1 and U1 feeding treatments, respectively, and was comparable between that treatments which indicated a significant increase in feeding of U1 treatment though the values were within the upper normal limit reported by Kaczmarowski *et al*. [11] with blood glucose concentration of healthy cows (2.54±0.88 mmol/L). The blood glucose concentration is one of the biochemical indicators by which one may conclude about body energy supply. In addition, there was no significant difference in serum total protein supplemented by different concentrate levels and energy sources. In male buffalo calves consuming wheat straw and concentrate mixture, Kumar and Dass [12] also reported non-significant effect of niacin supplementation on serum glucose level, serum total protein, albumin, and globulin in blood biochemical profiles. Albumin reflects long-term protein status, and plasma albumin levels could be altered by the effect of liver function, protein, and energy intake, age, and protein losses during certain diseases such as parasitism. Moreover, the concentration of total protein, globulin, albumin, and urea-N in blood serum is indicators of the adequacy or inadequacy of nitrogen in the diet of animals [13]. In this study, albumin levels and globulin were similar to those reported by Alves *et al*. [14] in dairy cows.

Inside the body of animals, cholesterol is synthesized from fatty acids and its concentration in the serum reflects body fat metabolism. In the present study, the availability of cholesterol in blood serum was found significantly different between SB2 and basal treatment (p<0.01). This was probably due to the higher intake of fibrous diets in basal treatment which is responsible for lowering the cholesterol content in serum [15]. In addition, serum proteins constitute a portion of the amino acid pool in the body, and it is believed to be indicative of the nutritional status of the animal. Total protein level in soybean treatment was lower (p<0.05) than that in the treatment with fish oil supplementation when supplemented with different energy sources. The different concentration of total protein in blood serum between SB2 and basal treatment was probably due to the difference in the amount of protein supplemented in the diets [2].

## Conclusion

Different protein sources affected the concentration of blood glucose, bilirubin, cholesterol, total protein and globulin, and different levels of concentrate, soybean oil, and fish oil contributed to the changes in levels of uric acid and protein in cattle blood profiles.

## Authors’ Contributions

NTN contributed to the design of the study. NHX and HTL conducted the data collection and analysis. NHX and HTL prepared the first draft and NTN did the correction of the manuscript. All authors have read and approved the final manuscript.
